# The impact of physical exercise with additional visual tasks on UDVA and accommodation sensitivity in children: the mediating role of kinetic visual acuity

**DOI:** 10.3389/fpubh.2024.1467651

**Published:** 2024-12-06

**Authors:** Miyu Wang, Guiming Zhu, Yihua Li, Pengfei Li, Haijie Shi, Limei Jiang, Yucui Diao, Rongbin Yin

**Affiliations:** School of Physical Education, Soochow University, Suzhou, China

**Keywords:** physical exercise, children, kinetic visual acuity, uncorrected distance visual acuity, accommodative sensitivity

## Abstract

**Purpose:**

This study investigates the impact of physical exercise supplemented with visual tasks on children’s uncorrected distance visual acuity (UDVA), kinetic visual acuity (KVA), and accommodative sensitivity, with an analysis of the mediating role of KVA.

**Methods:**

A total of 168 third-grade students from four natural classes in a primary school in Suzhou City were randomly assigned to either an experimental group (*n* = 86) or a control group (*n* = 82). The experimental group engaged in 30 cycles of ciliary muscle training tasks with visual targets presented for 3 s during physical exercises, while the control group participated in regular physical exercises. The intervention lasted 16 weeks, during which KVA, UDVA, and accommodative sensitivity of children were measured before and after the intervention using a kinetic visual acuity tester, a standard logarithmic visual acuity chart lightbox, and a lens flipper.

**Results:**

Post-intervention, the experimental group showed significant improvements in KVA, UDVA, and accommodative sensitivity (*p* < 0.05). The control group did not show significant changes in KVA and UDVA (*p* > 0.05), but did exhibit a significant improvement in accommodative sensitivity (*p* < 0.05). Additionally, children in the experimental group demonstrated significantly higher levels of KVA, UDVA, and accommodative sensitivity compared to the control group after the intervention (*p* < 0.05). A moderate positive correlation was found between KVA and both UDVA and accommodative sensitivity. KVA partially mediated the effect of additional visual tasks during physical exercise on UDVA in children (left eye 95% CI: 0.011—0.180; right eye 95% CI: 0.023—0.167). Moreover, KVA partially mediated the effect of additional visual tasks during physical exercise on accommodative sensitivity (95% CI: 0.021—0.245).

**Conclusion:**

Incorporating additional visual tasks into physical exercise effectively enhances KVA, UDVA, and accommodative sensitivity in children. There is a significant positive correlation between KVA and UDVA as well as between KVA and accommodative sensitivity. These visual tasks directly impact UDVA and accommodative sensitivity and indirectly influence them through the mediating effect of KVA.

## 1 Introduction

Eye health is a crucial component of national health, significantly affecting learning, life, work, and sustainable development. Currently, the global myopia situation is concerning, with the prevalence continuing to rise, and the myopia problem among adolescents around the world becoming increasingly severe. Research indicated that myopia has already affected nearly 30% of the world’s population, and it is projected to rise to 50% by 2050 (1). The top 10 countries with the highest myopia rates globally include China, Japan, South Korea, Singapore, Taiwan, Hong Kong, Malaysia, Thailand, the Philippines, and India. In these countries, myopia rates generally exceed 70%, with China and Singapore facing the most severe myopia problems (2). At present, the myopia rate among Chinese adolescents ranks first in the world. According to monitoring data from the National Health Commission of China in July 2021, the overall myopia prevalence rate among children and adolescents is 52.7%. The rates in primary school, middle school, and high school are 35.6, 71.1, and 80.5%, respectively. The widespread prevalence of myopia has become a significant factor in the decline of eye health among Chinese children and adolescents, posing a challenge to the vision of building a Healthy China.

In response, the Central Committee of the Communist Party of China and the State Council have issued various guidance documents, including the “Key Work Plan for Comprehensive Prevention and Control of Myopia in Children and Adolescents in 2022,” which emphasizes vision protection through physical activities. Research indicated that visual development varies among different age groups, with UDVA development mainly occurring before age 6 and KVA being most sensitive between ages 7 and 8. As children and adolescents aged 9–14 grow older, their visual acuity tends to decline significantly (11). Notably, third-grade students are at a sensitive stage for both static and dynamic vision development, with environmental factors contributing to a gradual increase in myopia prevalence. Therefore, this study focused on third-grade students as the research subjects.

Physical exercise is widely acknowledged as a critical measure for preventing and managing myopia among children and adolescents. Yurova et al. (3) conducted an intervention study indicating that regular physical exercise over 12 months can reduce the risk of myopia in children and adolescents. For those with mild to moderate myopia, physical exercise helps maintain existing vision and slows the progression of myopia. Giloyan et al. (4) found that increasing outdoor activity time can effectively intervene in the occurrence and progression of myopia. Engaging in at least 1 h of outdoor physical exercise daily can significantly slow down the progression of myopia. Mike et al. (5) concluded that regular physical exercise positively impacts vision development and that maintaining this routine over time can effectively reduce the likelihood of developing myopia. He et al. (6) found that physical exercise is an effective means of lowering myopia rates, with increased outdoor activity time contributing to a reduction in myopia incidence. Khalid et al. (7) suggested that compared to pharmacological, surgical, or optical interventions, implementing physical exercise interventions is more effective in enhancing the regulatory frequency of the ciliary muscles, strengthening neural regulation, and alleviating psychological stress. This approach is particularly beneficial for promoting the overall visual health of children and adolescents. Cai (8) emphasized the importance of kinetic visual acuity (KVA) in children’s and adolescents’ physical activities. The ability of physical exercise to enhance ciliary muscle accommodation is largely due to the involvement of KVA in most sports activities, with KVA reflecting ciliary muscle accommodation function. Additionally, there is a significant correlation between KVA and uncorrected distance visual acuity (UDVA), with KVA demonstrating a degree of predictive validity for UDVA. Furthermore, KVA serves as a mediating factor in the improvement of UDVA through physical exercise among children and adolescents. Accommodation sensitivity, a critical accommodative parameter, closely relates to myopia development and progression, serving as a sensitive indicator for predicting refractive errors (9). Poorer accommodation sensitivity is linked to faster progression of myopia. Therefore, this study investigated the impact of physical exercise with additional visual tasks on UDVA, KVA, and accommodation sensitivity in children. Additionally, it analyzed the mediating effect of KVA in these relationships.

## 2 Materials and methods

### 2.1 Participants

This study involved 168 third-grade students randomly selected from four natural classes in an elementary school in Suzhou City. Each pair of classes constituted a group, which was then randomly assigned as either an experimental or control group. The experimental study was conducted in the Physical Education and Health program. The study implemented a 16-week physical education intervention focusing on the experimental group, which included additional visual tasks, while the control group followed the standard physical education curriculum. There were no differences in KVA, UDVA, and accommodation sensitivity between the two groups, making them suitable for the experiment. The specific distribution of study participants is shown in Table 1.

**Table 1:** Distribution of study participants (*N* = 168).

Group	Male	Female	Total
Experimental group	42	44	86
Control group	40	42	82
Total	82	86	168

Participant selection criteria were as follows: (1) No prior use of orthokeratology lenses; (2) Absence of pathological eye diseases; (3) Normal cognitive and motor functions. The study protocol was in accordance with the Helsinki Declaration and was approved by the Ethics Committee of Suzhou University (No. SU-DA20201010H01).

### 2.2 Experimental methods

#### 2.2.1 Visual standard size

The visual standard size in this study was determined based on the actual visual acuity levels of the children, following the specifications of the standard logarithmic visual acuity chart. Adjustments were made based on participants’ average UDVA. For instance, a visual acuity of 4.9 on the logarithmic chart corresponded to a visual standard size of 9.16 millimeters. Given potential environmental and participant-specific variations, adjustments were flexibly applied as needed. Throughout the experiment, the visual standard size varied with distance, with the far visual standard set consistently at 3 meters to account for diverse influencing factors.

#### 2.2.2 Presentation frequency and duration of visual standards

Zhou et al. (10) found that in ciliary muscle exercises, visual standards presented for 1 s and 3 s are more effective than those presented for 5 s. Yin et al. (11) integrated ciliary muscle regulation training into physical education classes, grouping participants by frequencies of 15, 30, and 60 for visual intervention training. Results indicated that 30 sessions of visual far-near alternating recognition tasks per class had the most significant impact on improving children’s KVA and UDVA. Therefore, in this study, the visual standard presentation frequency was set at no less than 30 times per class, with a presentation duration of 3 s.

#### 2.2.3 Experimental design

The intervention was based on the “Compulsory Education Physical Education and Health Curriculum Standards (2022 Edition)” for second-stage students (12), and in conjunction with the semester teaching plan of physical education and health at a primary school in Suzhou, appropriate physical education content suitable for second-stage students was selected. The intervention of physical exercise with additional visual tasks was designed primarily around the principles of ciliary muscle regulation and essential movement skills. During the teaching process, specific instructional content was integrated with the principles of ciliary muscle training. Considering the physical and mental development characteristics of children, it was decided to primarily focus on teaching basketball within ball sports (Table 2). The teaching process was structured around four components: learning, practicing, competing, and evaluating, with ciliary muscle training integrated as a fundamental part of physical education classes and included in a 10-min physical exercise session. The experimental group completed 30 sessions of ciliary muscle training with a visual standard presentation duration of 3 s during physical education classes. The control group underwent regular physical education instruction without additional visual tasks. Both groups were standardized in terms of sports facilities, duration, and activities. To mitigate the impact of extraneous variables, the same individual conducted physical education classes for all four classes from the beginning to the end of the experiment. Additionally, children in both groups were advised to minimize the use of electronic devices after classes to reduce eye strain.

**Table 2:** Experimental design.

Category	Activity	Exercise content	Exercise time	Exercise frequency	Duration of ciliary muscle intervention
Open motor skills	Basketball	Passing and catching exercises, rebounding, stationary dribbling with high and low movements, stationary dribbling with change of direction toward markers, dribbling in a straight line between markers, two-handed chest pass and catch, and relay shooting off the backboard, among others.	16 weeks in total, with each session lasting 40 min	Three times a week	The experimental group is required to complete 30 sessions of ciliary muscle training with a visual standard presentation duration of 3 s during physical education classes. The control group undergoes regular physical education instruction without the requirement of visual recognition tasks.

During the experiment, two main exercise methods were employed: Firstly, teachers or group leaders held visual standard cards, ensuring a consistent distance of 3 meters from the students. After each identification of the visual standard card, students maintained their gaze for 3 s before lowering their focus. Secondly, visual standards were affixed to sports equipment. Under teacher guidance, students practiced at a distance of 3 meters from the equipment using the same method.

### 2.3 Testing methods

#### 2.3.1 UDVA test

A standard logarithmic visual acuity chart lightbox was used as the testing tool. During data collection, participants stood 5 meters away from the lightbox and underwent tests for each eye sequentially. The test administrator used a rod to point to the “E” optotype on the visual acuity chart, and participants had to verbally state or gesture the direction of the gap in the optotype. Testing started from the largest “E” optotype at the top of the chart and proceeded downward row by row until the smallest optotype that the participant could clearly identify was found. The corresponding visual acuity value of that optotype size was recorded as the participant’s unaided far vision. If participants wore glasses or contact lenses, they were asked to remove them prior to the unaided far vision measurement.

#### 2.3.2 KVA test

The XP.14-TDJ905 kinetic visual acuity tester, manufactured by Shanghai Hump Automation Technology Co., Ltd., was used in accordance with the national standard GB18463-2001. When the device is activated and in testing mode, a dynamic “C”-shaped optotype appears in the eyepiece. This optotype gradually approaches the participant from a distance, with the gap direction randomly changing (upwards, downwards, leftwards, or rightwards). Participants had to accurately determine the direction of the gap in the optotype as quickly as possible and promptly respond using a control lever (13). After each test, the tester automatically displayed the participant’s KVA value. The test administrator promptly recorded this value and pressed the reset button to prepare for the next test. The average of three recorded values was taken as the participant’s final KVA value.

#### 2.3.3 Accommodation sensitivity test

The main testing tools included a stopwatch, flipper, and an accommodation sensitivity test reading card. To ensure data accuracy, tests were conducted in a well-lit and quiet classroom. Participants held the flipper with the +2.00D lens in front of both eyes and identified the “E” optotype on the reading card at 40 cm. They indicated the direction of the gap in the “E” optotype, then switched to the −2.00D lens. After clearly seeing the optotype again, they switched back to the +2.00D lens, repeating this process. The cycle was timed for 1 min, and the number of flips completed in that time was recorded (14).

### 2.4 Statistical analysis

Data entry and organization were conducted using Excel 2021, while SPSS 26.0 was employed for comprehensive data analysis. All data results were presented as mean ± standard deviation (M ± SD). Firstly, descriptive statistics were performed on the relevant test indicators of the experimental subjects. Secondly, an independent samples *t*-test was used to test the homogeneity of the two groups’ data. A 2 (Time: pre-test, post-test) × 2 (Group: experimental group, control group) mixed-design analysis of variance (ANOVA) was then conducted to compare the KVA, UDVA, and accommodation sensitivity within and between the two groups of children. Finally, Pearson correlation analysis was employed for pairwise correlation analysis, and mediation effects were tested using the Bootstrap method based on SPSS 26.0 Process macro.

## 3 Results

### 3.1 Changes in KVA, UDVA, and accommodation sensitivity of children in both groups before and after the experiment

A 2 × 2 mixed-design analysis of variance (ANOVA) was conducted to investigate the effects of additional visual task exercises during physical education on the KVA, UDVA, and accommodation sensitivity of children (Tables 3, 4). Changes in vision and accommodation sensitivity were observed among children.

**Table 3:** Within-group comparisons of relevant test indicators for two groups of children before and after the experiment.

Group	Indicators	Pre-test (I)	Post-test (J)	Mean difference (I-J)	*F* value	*p*-value	Partial Eta squared
Experimental group	KVA	0.309 ± 0.22	0.401 ± 0.23	−0.093	23.303	0.000	0.123
	UDVA (left eye)	4.905 ± 0.24	4.979 ± 0.23	−0.074	34.173	0.000	0.171
	UDVA (right eye)	4.916 ± 0.23	4.981 ± 0.24	−0.065	26.259	0.000	0.137
	Accommodation sensitivity	6.593 ± 1.70	9.244 ± 2.08	−2.651	188.093	0.000	0.531
Control group	KVA	0.321 ± 0.20	0.323 ± 0.20	−0.002	0.007	0.934	0.000
	UDVA (left eye)	4.899 ± 0.27	4.900 ± 0.23	−0.001	0.009	0.926	0.000
	UDVA (right eye)	4.885 ± 0.27	4.887 ± 0.26	−0.002	0.009	0.925	0.000
	Accommodation sensitivity	6.494 ± 1.63	8.293 ± 1.63	−1.779	82.561	0.000	0.332

* indicates significant difference (*p* < 0.05).

**Table 4:** Between-group comparisons of relevant test indicators for two groups of children before and after the experiment.

Indicators	Measurement time	Group (I)	Group (J)	Mean difference (I-J)	*F* value	*p*-value	Partial eta squared
KVA	Pre-test	Experimental group	Control group	−0.012	0.136	0.713	0.001
	Post-test	Experimental group	Control group	0.079	5.770	0.017	0.034
UDVA (left eye)	Pre-test	Experimental group	Control group	0.006	0.022	0.883	0.000
	Post-test	Experimental group	Control group	0.079	4.807	0.030	0.028
UDVA (right eye)	Pre-test	Experimental group	Control group	0.031	0.626	0.430	0.004
	Post-test	Experimental group	Control group	0.095	5.851	0.017	0.034
Accommodation sensitivity	Pre-test	Experimental group	Control group	0.099	0.148	0.701	0.001
	Post-test	Experimental group	Control group	0.952	10.805	0.001	0.061

* indicates significant difference (*p* < 0.05).

A 2 (Time: pre-test, post-test) × 2 (Group: experimental, control) mixed-design analysis of variance (ANOVA) was conducted to analyze the KVA of participants. The results revealed significant main effects for time (*F*(1,166) = 11.776, *p* < 0.05) and a significant time × group interaction effect (*F*(1,166) = 10.979, *p* < 0.05). Simple effects analysis indicated a significant improvement in KVA in the experimental group from pre-test to post-test (*F*(1,166) = 23.303, *p* < 0.05), while the control group showed a slight, non-significant improvement (*F*(1,166) = 0.007, *p* > 0.05). No significant difference in KVA was found between the two groups before the experiment (*F*(1,166) = 0.136, *p* > 0.05), whereas significant differences emerged after the experiment (*F*(1,166) = 5.770, *p* < 0.05).

A 2 (Time: pre-test, post-test) × 2 (Group: experimental, control) mixed-design analysis of variance (ANOVA) was conducted to analyze the uncorrected distance visual acuity (UDVA) of participants. The results showed significant main effects for time (Left eye: *F*(1,166) = 17.231, *p* < 0.05; Right eye: *F*(1,166) = 13.301, *p* < 0.05) and significant time × group interaction effects (Left eye: *F*(1,166) = 16.137, *p* < 0.05; Right eye: *F*(1,166) = 12.341, *p* < 0.05). Simple effects analysis revealed a significant improvement in UDVA in the experimental group from pre-test to post-test (Left eye: *F*(1,166) = 34.173, *p* < 0.05; Right eye: *F*(1,166) = 26.259, *p* < 0.05), while the control group showed a slight, non-significant improvement (Left eye: *F*(1,166) = 0.009, *p* > 0.05; Right eye: *F*(1,166) = 0.009, *p* > 0.05). No significant difference in UDVA was found between the two groups before the experiment (Left eye: *F*(1,166) = 0.022, *p* > 0.05; Right eye: *F*(1,166) = 0.626, *p* > 0.05), whereas significant differences emerged after the experiment (Left eye: *F*(1,166) = 4.807, *p* < 0.05; Right eye: *F*(1,166) = 5.851, *p* < 0.05).

A 2 (Time: pre-test, post-test) × 2 (Group: experimental, control) mixed-design analysis of variance (ANOVA) was conducted to analyze the accommodation sensitivity of participants. The results revealed significant main effects for time (*F*(1,166) = 258.651, *p* < 0.05) and a significant time × group interaction effect (*F*(1,166) = 9.490, *p* < 0.05). Simple effects analysis indicated a significant improvement in accommodation sensitivity from pre-test to post-test in both the experimental group (*F*(1,166) = 188.093, *p* < 0.05) and the control group (*F*(1,166) = 82.561, *p* < 0.05). No significant difference in accommodation sensitivity was found between the two groups before the experiment (*F*(1,166) = 0.148, *p* > 0.05), whereas significant differences emerged after the experiment (*F*(1,166) = 10.805, *p* < 0.05).

### 3.2 Correlation analysis of KVA, UDVA, and accommodation sensitivity in children

Pearson correlation analysis (Table 5) indicated significant correlations between KVA, UDVA, and accommodation sensitivity (all *p* < 0.05). Specifically, KVA was moderately positively correlated with UDVA in the left eye (*r* = 0.527, *p* < 0.01) and right eye (*r* = 0.496, *p* < 0.01), as well as with accommodation sensitivity (*r* = 0.517, *p* < 0.01).

**Table 5:** The relationship between KVA, UDVA, and accommodation sensitivity in children.

Indicators	KVA	UDVA (left eye)	UDVA (right eye)	Accommodation sensitivity
KVA	1	0.527^**^	0.496^**^	0.517^**^
UDVA (left eye)		1	0.741^**^	0.366^**^
UDVA (right eye)			1	0.266^*^
Accommodation sensitivity				1

* indicates significant difference (*p* < 0.05), ** indicates *p* < 0.01.

### 3.3 Mediation model testing of the impact of additional visual task exercises in physical education on UDVA and accommodation sensitivity in children

To examine whether kinetic visual acuity (KVA) mediates the effect of additional visual task exercises during physical education on uncorrected distance visual acuity (UDVA) and accommodation sensitivity in elementary school students, we first constructed a binary variable based on group assignment. The group engaged in additional visual tasks was coded as 1, while the group participating in regular physical education was coded as 0. We then employed the SPSS 26.0 Process macro (Model 4) to perform a mediation analysis using the bootstrap method with a 95% confidence interval. A coefficient was deemed non-significant if the confidence interval includes 0; otherwise, it was considered significant.

In this study, the binary variable served as the independent variable, KVA as the mediating variable, and UDVA or accommodation sensitivity as the dependent variables. The bootstrap analysis was conducted with 5,000 iterations of random sampling with replacement. The independent variable was defined as the engagement in additional visual task exercises during physical education, coded as “1” for the experimental group and “0” for the control group. KVA was measured as the difference between post-test and pre-test scores (ΔKVA = post-test score-pre-test score). Similarly, UDVA (dependent variable 1) was operationalized as the difference in scores for the left eye (ΔUDVA = post-test score-pre-test score), and accommodation sensitivity (dependent variable 2) was measured as the difference between post-test and pre-test scores (Δ accommodation sensitivity = post-test score-pre-test score).

#### 3.3.1 Mediation analysis of KVA in the impact of additional visual task exercises in physical education on UDVA

The regression analysis results indicated that additional visual task exercises during physical education significantly predict uncorrected distance visual acuity (UDVA) in the left eye (*β* = 0.594, *p*<0.001). When kinetic visual acuity (KVA) was included in the regression model, additional visual task exercises significantly positively predicted KVA (*β* = 0.497, *p*<0.01), and KVA significantly positively predicted left eye UDVA (*β* = 0.162, *p*<0.05). In this context, additional visual task exercises still significantly positively predicted left eye UDVA (*β* = 0.514, *p*<0.001).

Similarly, additional visual task exercises significantly positively predicted right eye UDVA (*β* = 0.525, *p*<0.001). When KVA was included in the model, additional visual task exercises significantly positively predicted KVA (*β* = 0.497, *p*<0.01), and KVA significantly positively predicted right eye UDVA (*β* = 0.174, *p*<0.05). In this scenario, additional visual task exercises still significantly positively predicted right eye UDVA (*β* = 0.438, *p*<0.01).

**Figure 1:**
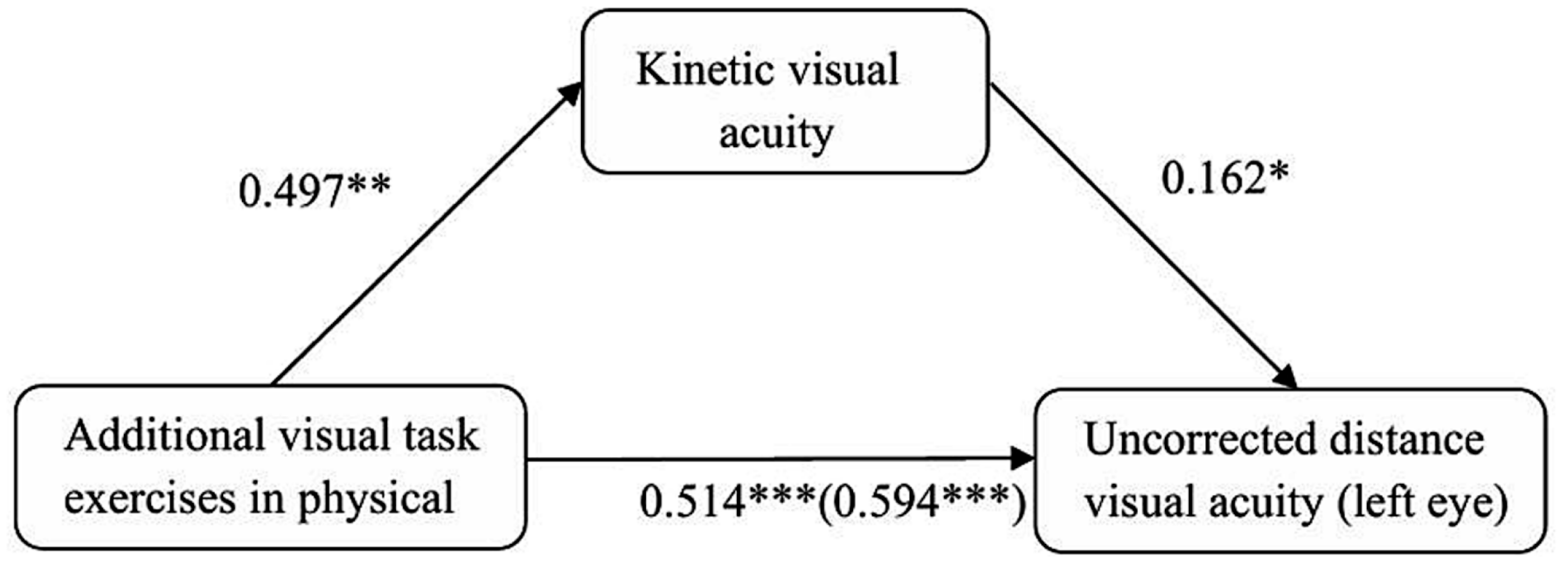
The diagram of the mediation effect model of kinetic visual acuity between physical exercise and uncorrected distance visual acuity in the left eye.

**Figure 2:**
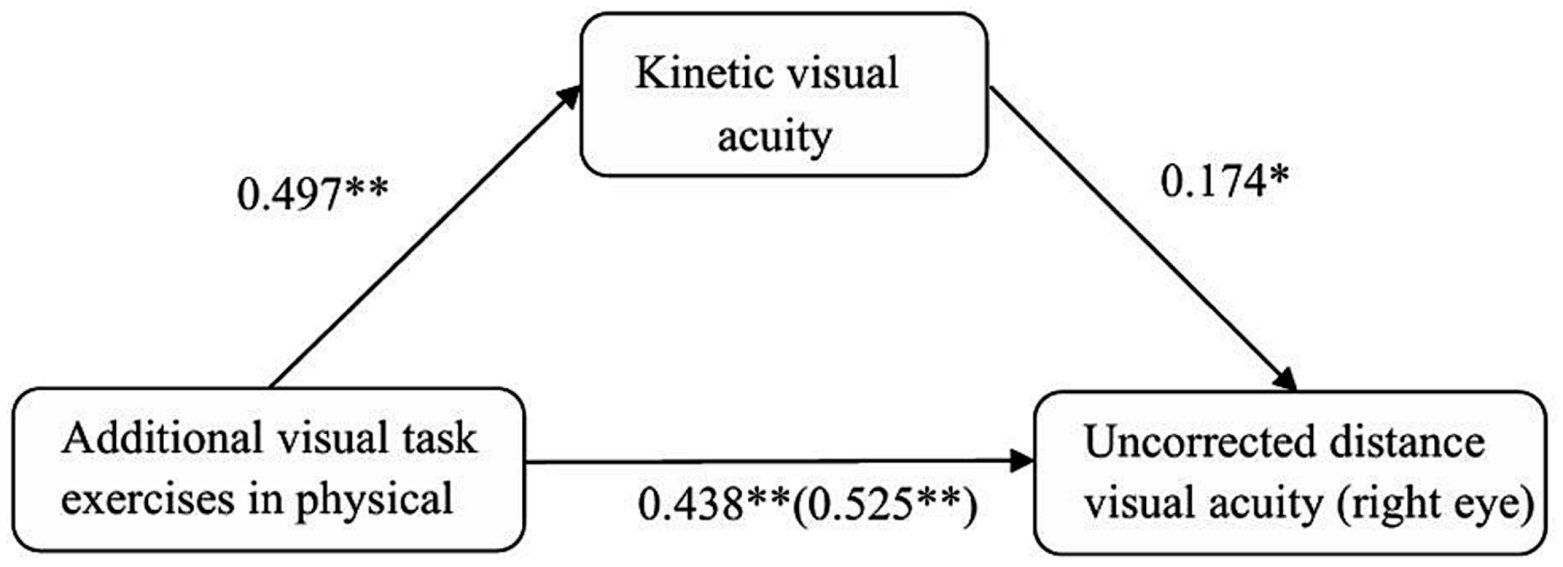
Path diagram testing the mediation effect of kinetic visual acuity between physical exercise and uncorrected distance visual acuity in the right eye.

Mediation analysis results (Table 6 and Figures 1, 2) revealed a significant mediating effect of KVA in the relationship between additional visual task exercises and left eye UDVA. The direct effect was 0.514 (95% CI [0.027, 0.100], excluding 0), which was significant and accounted for 87% of the total effect of additional visual task exercises on left eye UDVA (total effect size = 0.594). The indirect effect was 0.080 (95% CI [0.011, 0.180], excluding 0), which was also significant and accounted for 13% of the total effect, indicating a partial mediating role of KVA.

**Table 6:** Effect size analysis of additional visual task exercises in physical education, KVA, and UDVA.

Indicators	Effect relationships	Effect	Bootstrap 95% CI		Proportion of effect
			LLCI	ULCI	
UDVA (left eye)	Total effect	0.594	0.037	0.109	
	Direct effect	0.514	0.027	0.100	87%
	Indirect effect	0.080	0.011	0.180	13%
UDVA (right eye)	Total effect	0.525	0.028	0.100	
	Direct effect	0.438	0.017	0.090	83%
	Indirect effect	0.087	0.023	0.167	17%

For right eye UDVA, KVA also exhibited a significant mediating effect. The direct effect was 0.438 (95% CI [0.017, 0.090], excluding 0), accounting for 83% of the total effect of additional visual task exercises on right eye UDVA (total effect size = 0.525). The indirect effect was 0.087 (95% CI [0.023, 0.167], excluding 0), accounting for 17% of the total effect, again indicating a partial mediating role of KVA in this relationship.

#### 3.3.2 Testing the mediating effect of KVA in the impact of additional visual tasks during sports exercise on accommodation sensitivity

The regression analysis results indicated that additional visual task exercises in physical education significantly positively predicted accommodation sensitivity (*β* = 0.477, *p* < 0.01). Furthermore, after including kinetic visual acuity (KVA) in the regression model, additional visual task exercises still significantly positively predicted KVA (*β* = 0.497, *p* < 0.01), and KVA significantly positively predicted accommodation sensitivity (*β* = 0.232, *p* < 0.01). At this stage, additional visual task exercises continued to significantly positively predict accommodation sensitivity (*β* = 0.362, *p* < 0.05).

**Figure 3:**
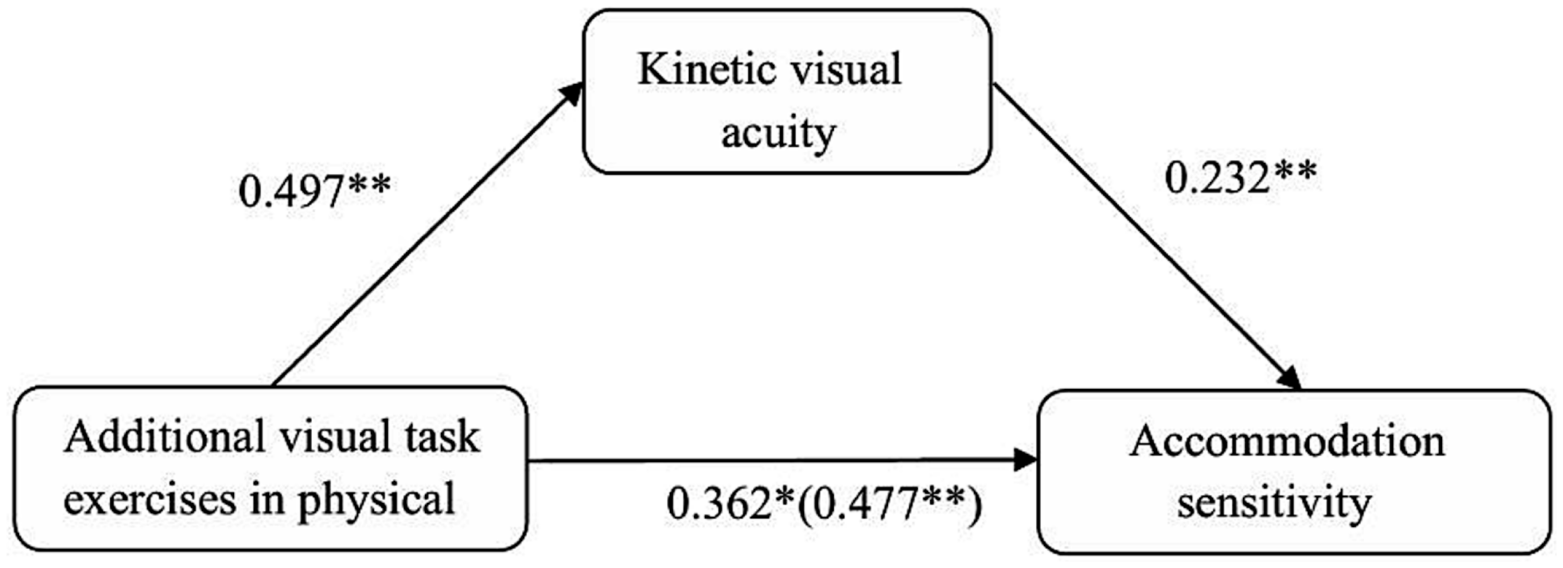
Path diagram of the mediation effect of KVA between physical exercise and accommodation sensitivity.

The mediation analysis results (Table 7 and Figure 3) showed that KVA had a significant mediating effect between additional visual task exercises and accommodation sensitivity. The direct effect was 0.362, with a 95% confidence interval (CI) of [0.115, 1.215], which did not include 0, indicating a significant direct effect. This direct effect accounted for 76% of the total effect of additional visual task exercises on accommodation sensitivity (total effect value = 0.477). The indirect effect was 0.115, with a 95% CI of [0.021, 0.245], which also did not include 0, indicating a significant indirect effect. This indirect effect accounted for 24% of the total effect, suggesting that KVA partially mediated the impact of additional visual task exercises on accommodation sensitivity.

**Table 7:** Effect size analysis of physical exercise with additional visual tasks, KVA, and accommodation sensitivity.

Effect relationships	Effect	Bootstrap 95% CI		Effect proportion
		LLCI	ULCI	
Total effect	0.477	0.331	1.423	
Direct effect	0.362	0.115	1.215	76%
Indirect effect	0.115	0.021	0.245	24%

## 4 Discussion

### 4.1 The impact of physical exercise with additional visual tasks on KVA in children

This study demonstrated a significant improvement in KVA among children in the experimental group following the intervention, while the control group showed only slight, non-significant improvements. This indicated that physical exercise with additional visual tasks is more effective in enhancing KVA in children, supporting this study’s hypothesis. Additionally, it is important to acknowledge that physical exercise also helps maintain existing levels of visual acuity. Hoshina et al. (15) measured static and kinetic visual acuity in 102 Japanese male professional baseball players, revealing that engagement in sports activities can improve or enhance KVA. Consistent with this, research suggested that children who regularly engage in sports activities exhibit higher KVA levels compared to those who do not or only occasionally participate in sports (16). This finding aligned with comparative studies on sports such as baseball (17) and table tennis (18), further supporting this study’s viewpoint. Cao et al. (19) demonstrated that physical exercise with additional visual tasks effectively improves students’ visual conditions and maintains visual health, enhancing KVA levels among 6-9-year-old students. Zhou et al. (20) investigated vision changes among fourth-grade students by incorporating KVA recognition tasks into physical activities. They found that adding KVA recognition tasks during regular physical exercise enhanced students’ visual tracking abilities by having them continuously observe the trajectory of objects, which effectively engages the ciliary muscles to contract or relax, thereby enhancing visual tracking capabilities. Wylegala (21) also suggested that physical exercise with visual recognition tasks can increase the frequency of ciliary muscle regulation, improve eye muscle coordination, reduce intraocular pressure, and significantly enhance KVA levels.

Therefore, compared to traditional ciliary muscle regulation training, incorporating ciliary muscle training with a frequency of 30 cycles and a stimulus presentation time of 3 s during physical exercise can effectively alleviate the tension caused by prolonged ciliary muscle activity. This enhancement in ciliary muscle regulation facilitates optimal development of visual acuity among children, particularly in elevating KVA levels.

### 4.2 The impact of physical exercise with additional visual tasks on UDVA in children

In this study, we implemented a physical exercise intervention incorporating visual recognition tasks with a frequency of 30 cycles and a stimulus presentation time of 3 s. Visual stimuli were either held by teachers or attached to objects such as basketballs, cones, and poles. The results showed a significant improvement in uncorrected distance visual acuity (UDVA) among children in the experimental group compared to their pre-intervention levels. Research by Zhang et al. (22) demonstrated that integrating alternating visual recognition tasks into cheerleading instruction effectively alleviated ciliary muscle tension, enhanced their regulatory capacity, and subsequently improved UDVA among elementary school students. This supported the feasibility of long-term physical exercise with additional visual tasks, aligning with the developmental characteristics of elementary students’ vision. Similarly, Cao et al. (19) found that physical exercise with additional visual tasks strengthened the regulatory function of the ciliary muscles, alleviating muscle tension and effectively improving UDVA among children aged 6–9. Muñoz et al. (23) highlighted the positive effects of judo and karate on controlling the decline in kinetic visual acuity (KVA), indicating their efficacy in enhancing UDVA as essential means of physical fitness. Morgan et al. (24) noted that schools, as primary learning environments, often impose significant academic pressure and provide insufficient outdoor physical activity time, leading to prolonged near-work activities. This prolonged engagement places the ciliary muscles under sustained tension, disrupting their regulatory function and resulting in decreased visual acuity. However, integrating visual recognition tasks into physical activities can effectively stimulate the ciliary muscles as children track moving objects, increasing their regulatory frequency and aiding in the restoration of ciliary muscle function, thereby improving UDVA among children.

Therefore, compared to traditional ciliary muscle regulation training, integrating visual recognition tasks with a frequency of 30 cycles and a stimulus presentation time of 3 s into physical exercise can more effectively engage the contraction and relaxation of the ciliary muscles in children. This regimen exercises the regulatory function of the ciliary muscles, preventing a decrease in their regulatory capacity due to spasms and thereby promoting the development of UDVA among children.

### 4.3 The impact of physical exercise with additional visual tasks on accommodation sensitivity in children

This study revealed a significant improvement in accommodation sensitivity among both the experimental and control group children post-intervention. However, the experimental group exhibited a greater increase in accommodation sensitivity compared to the control group. This finding indicated that physical exercise with additional visual tasks had a more pronounced and effective impact on children’s accommodation sensitivity than traditional physical exercise, aligning with this study’s hypothesis. This outcome can be attributed to the integration of visual recognition tasks into the physical exercise regimen, with a frequency of 30 cycles and a stimulus presentation time of 3 s. This approach provided substantial exercise for the regulatory function of the ciliary muscles and involved continuous practice of near-far vision alternation, a component lacking in conventional physical exercise. Consequently, the control group showed a smaller increase in accommodation sensitivity compared to the experimental group. Supporting this, Diao (25) found that table tennis can improve students’ accommodation sensitivity by enhancing the tracking ability of the extraocular muscles, thereby engaging the contraction and relaxation of the ciliary muscles and delaying the onset and progression of myopia. Similarly, Chu (34) proposed table tennis as an effective means of improving accommodation sensitivity in children and adolescents.

Both visual acuity and accommodation sensitivity are malleable. Accommodation sensitivity is closely associated with visual development and may decline with age. Research by Lei et al. (26) found that accommodation sensitivity tended to be lower in younger primary school children, particularly those around 6 years old, and relatively higher in older primary school children. They speculated that this phenomenon could be attributed to the age-related development of the eyeball, which enlarges and elongates with increasing age. Therefore, during the primary school years, it is crucial to screen for and identify children with abnormal accommodation sensitivity and provide timely and targeted interventions to delay the progression of myopia to some extent.

### 4.4 The relationship between KVA, UDVA, and accommodation sensitivity in children

Based on Pearson correlation analysis, significant correlations were found among kinetic visual acuity (KVA), uncorrected distance visual acuity (UDVA), and accommodation sensitivity in children. Specifically, KVA showed a significant positive correlation with both UDVA and accommodation sensitivity. Scialfa et al. (27) also noted a correlation between KVA and UDVA, although they cautioned that using UDVA to predict KVA may introduce bias. Similarly, Freeman et al. (28) found that KVA can serve as a predictor for UDVA, suggesting that poorer KVA may lead to greater difficulty in daily near-distance visual activities, which aligns with the findings of this study. Additionally, these results revealed a significant positive correlation between KVA and accommodation sensitivity, consistent with the findings of Zhou et al. (10). Zhou and colleagues proposed that ciliary muscle training can optimize the regulatory function of the ciliary muscles, thereby strengthening KVA and enhancing accommodation sensitivity. This suggested that interventions aimed at improving ciliary muscle function could be beneficial for enhancing both KVA and accommodation sensitivity in children.

### 4.5 The mediating role of KVA in the effect of physical exercise with additional visual tasks on UDVA in children

In this study, we utilized the Bootstrap method to investigate the mediating role of KVA in the effect of physical exercise with additional visual tasks on UDVA in children. The analysis indicated that physical exercise with additional visual tasks significantly influenced UDVA in these children. Furthermore, KVA was found to have a significant impact on UDVA. Notably, KVA partially mediated the relationship between physical exercise with additional visual tasks and UDVA, accounting for 13 and 17% of the effect in the left and right eyes, respectively. This suggested that physical exercise with additional visual tasks can directly affect UDVA and also indirectly influence UDVA through KVA.

The developmental sequence of visual function indicated that KVA can maintain and promote the development of UDVA, serving as a crucial predictor of UDVA (19). Durrie et al. (29) found that targeted KVA training can improve UDVA in individuals with myopia over a specific period. Sun et al. (30) demonstrated that both KVA and UDVA undergo changes with age, exhibiting consistent trends. Previous research had consistently shown that improvements in KVA often precede those in UDVA. It had been hypothesized that KVA may act as a mediating variable between physical exercise and UDVA, emphasizing its predictive value for UDVA levels. Thus, focusing on enhancing KVA during vision interventions and optimizing physical exercise plans based on KVA principles may be more effective in improving UDVA, consistent with the findings of this study. Therefore, incorporating additional visual tasks into physical education classes is essential to promote the development of both static and kinetic visual acuity in children and to delay the onset of myopia.

### 4.6 The mediating role of KVA in the effect of physical exercise with additional visual tasks on accommodation sensitivity in children

This study utilized the Bootstrap method to investigate the mediating role of kinetic visual acuity (KVA) in the relationship between physical exercise with additional visual tasks and accommodation sensitivity in children. The analysis revealed that physical exercise with additional visual tasks significantly influenced accommodation sensitivity in these children. Furthermore, KVA significantly affected accommodation sensitivity. KVA was found to partially mediate the relationship between physical exercise with additional visual tasks and accommodation sensitivity, with an indirect effect value of 0.115 and a mediation proportion of 24%. This suggested that physical exercise with additional visual tasks can directly impact accommodation sensitivity and also indirectly influence it through KVA.

Williams et al. (31) found that binocular visual acuity is more representative of everyday visual tasks than monocular visual acuity. KVA, as a functional aspect of vision, is closely associated with binocular visual acuity (32). Cai (8) suggested that the correlation between KVA and the functioning of the ciliary muscle can be attributed to the dynamic changes between near and far vision required in most physical exercises. Yuan et al. (33) demonstrated that improvement in KVA can alleviate ciliary muscle tension, thereby enhancing binocular accommodation sensitivity, a finding consistent with the results of this study. Therefore, emphasizing the integration of KVA tasks, such as those involving a 30 Hz frequency with a 3-s presentation duration, into physical exercise routines is essential to enhance children’s accommodation sensitivity. This approach can improve ciliary muscle function and promote visual health among children.

## 5 Conclusion

The integration of additional visual tasks into physical exercises can effectively enhance kinetic visual acuity (KVA), uncorrected distance visual acuity (UDVA), and accommodative sensitivity in children. Significant positive correlations were observed between KVA and UDVA, as well as between KVA and accommodative sensitivity. Moreover, KVA plays a partially mediating role in the impact of additional visual tasks integrated into physical exercises on UDVA and accommodative sensitivity among children.

## 6 Shortcomings and prospects

There are limitations in the sampling of this study. The subjects were selected from four natural classes of third graders in a primary school in Suzhou, totaling 168 students. Due to constraints in manpower, resources, and other factors, the sample size and representativeness do not reflect the entire population of children nationwide. Future research should have a broader and more standardized sampling scope. Additionally, the different effects of physical exercise with additional tasks compared to exercise combined with ciliary muscle training on children’s vision and accommodation sensitivity require further exploration and research.

## Data Availability

The raw data supporting the conclusions of this article will be made available by the authors, without undue reservation.

## References

[1] International Myopia Research Institute. Report on the impact of myopia by the international myopia research institute. Chin J Exper Ophthal. (2021) 39:1091–103. doi: 10.3760/cma.j.cn115989-20210623-00369

[2] Liang J Pu Y Chen J Liu M Ouyang B Jin Z. Global prevalence, trend and projection of myopia in children and adolescents from 1990 to 2050: a comprehensive systematic review and meta-analysis. Br J Ophthalmol. (2024) 24:bjo-2024-325427. doi: 10.1136/bjo-2024-325427 39317432

[3] Yurova OV Andjelova DV Chayka AA. The influence of physical loads on the functional parameters of the eyes in the children and adolescents regularly engaged in sports activities. Vopr Kurortol Fizioter Lech Fiz Kult. (2017) 94:44–8. doi: 10.17116/kurort201794344-48, PMID: 28884738

[4] Aida G Tsovinar H Varduhi P. Risk factors for developing myopia among schoolchildren in Yerevan and Gegharkunik Province, Armenia. Ophthalmic Epidemiol. (2017) 24:97–103. doi: 10.1080/09286586.2016.1257028, PMID: 28032802

[5] Yang M Luensmann D Fonn D Woods J Jones D Gordon K. Myopia prevalence in Canadian school children: a pilot study. Eye. (2018) 32:1042–7. doi: 10.1038/s41433-018-0015-5, PMID: 29391573 PMC5997685

[6] He M Xiang F Zeng Y Mai J Chen Q Zhang J. Effect of time spent outdoors at school on the development of myopia among children in China: a randomized clinical trial. JAMA. (2015) 314:1142–8. doi: 10.1001/jama.2015.10803, PMID: 26372583

[7] Khalid K Padda J Pokhriyal S Hitawala G Khan MS Upadhyay P. Pseudomyopia and its association with anxiety. Cureus. (2021) 13:e17411. doi: 10.7759/cureus.17411, PMID: 34589322 PMC8459808

[8] Cai G. Prevention and control of myopia in children and adolescents by physical exercise. Sport Sci Res. (2022) 43:18.

[9] Allen PM O’Leary DJ. Accommodation functions: co-dependency and relationship to refractive error. Vis Res. (2006) 46:491–505. doi: 10.1016/j.visres.2005.05.007, PMID: 16009391

[10] Zhou S Zhang M Zheng W Yin R Chen G. Effects of physical activity combined with different visual target presentation durations of ciliary-muscle training on visual acuity in children. Front Public Health. (2023) 11:1191112. doi: 10.3389/fpubh.2023.1191112, PMID: 37538276 PMC10394291

[11] Yin R Xu J Wang H Zhou S Zhang M Cai G. Effect of physical activity combined with extra ciliary-muscle training on visual acuity of children aged 10-11. Front Public Health. (2022) 10:949130. doi: 10.3389/fpubh.2022.949130, PMID: 36111187 PMC9468474

[12] Ji L. Interpretation of physical education and health curriculum standards for compulsory education (2022 edition) in China. China Sport Sci. (2022) 42:3–67. doi: 10.16469/j.css.202205001

[13] Jin G Pan J Cai G. Practical significance and empirical research on Students’Visual health through physical exercise. J Cap Univ Phys Educ Sport. (2021) 33:40–8. doi: 10.14036/j.cnki.cn11-4513.2021.01.005

[14] Ren Y Lu Z. Study on the curriculum of visual training. J Guangzhou Sport Univ. (2020) 40:95–100. doi: 10.13830/j.cnki.cn44-1129/g8.2020.05.021

[15] Hoshina K Tagami Y Mimura O. A study of static, kinetic, and dynamic visual acuity in 102 Japanese professional baseball players. Clin Ophthalmol. (2013) 7:627–32. doi: 10.2147/OPTH.S41047, PMID: 23569356 PMC3615904

[16] Ishigaki H Miyao M. Differences in dynamic visual acuity between athletes and nonathletes. Percept Mot Skills. (1993) 77:835–9. doi: 10.2466/pms.1993.77.3.835, PMID: 8284163

[17] Rouse MW DeLand P Christian R Hawley J. A comparison study of dynamic visual acuity between athletes and nonathletes. J Am Optom Assoc. (1988) 59:946–50. PMID: 3209790

[18] Jafarzadehpur E Yarigholi MR. Comparison of visual acuity in reduced lumination and facility of ocular accommodation in table tennis champions and non- players. J Sports Sci Med. (2004) 3:44–8. PMID: 24497820 PMC3896113

[19] Cao J Cai G Wang G. Effects of physical activities with visual tasks on kinetic and static visual acuity in children. Chin J Rehabil Theory Pract. (2019) 25:112–5. doi: 10.3969/j.issn.1006-9771.2019.01.016

[20] Zhou S Zhou C Tan Q. Effect of closed skills physical activity exercises with dynamic visual task on visual function for pupils with myopia at grade four at primary school. Chin J Rehabil Theory Pract. (2020) 26:1383–9. doi: 10.3969/j.issn.1006-9771.2020.12.003

[21] Wylegala A. The effects of physical exercises on ocular physiology: a review. J Glaucoma. (2016) 25:e843–9. doi: 10.1097/IJG.0000000000000454 27275655

[22] Zhang Y. Research on influence on Myope by visual tasks added cheerleading exercise. Suzhou, China: Soochow University (2020).

[23] Muñoz M Ballesteros S. Sports can protect dynamic visual acuity from aging: a study with young and older judo and karate martial arts athletes. Atten Percept Psychophys. (2015) 77:2061–73. doi: 10.3758/s13414-015-0901-x, PMID: 25893472

[24] Morgan IG French AN Ashby RS Guo X Ding X He M. The epidemics of myopia: aetiology and prevention. Prog Retin Eye Res. (2017) 62:134–49. doi: 10.1016/j.preteyeres.2017.09.004 28951126

[25] Diao Y. Experimental study of the effect of table tennis and badminton on myopia intervention in children. Guangzhou, China: Guangzhou Sport University (2021).

[26] Lei Z Tang J Jiang L. Observation of adjustment sensitivity in myopic adolescents. China J Prev Med. (2016) 28:723–5. doi: 10.19485/j.cnki.issn1007-0931.2016.07.022

[27] Scialfa CT Garvey PM Gish KW Deering LM Leibowitz HW Goebel CC. Relationships among measures of static and dynamic visual sensitivity. Hum Factors. (1988) 30:677–87. doi: 10.1177/001872088803000604 3235085

[28] Freeman EE Muñoz B Turano KA. Dynamic measures of visual function and their relationship to self-report of visual functioning. Invest Ophthalmol Vis Sci. (2006) 47:4762–6. doi: 10.1167/iovs.06-0436, PMID: 17065485

[29] Durrie D McMinn PS. Computer-based primary visual cortex training for treatment of low myopia and early presbyopia. Trans Am Ophthalmol Soc. (2007) 105:132–8. PMID: 18427602 PMC2258094

[30] Sun L Cai G Yin R. Correlation of static visual acuity and kinetic visual acuity in children and its implication to physical activity. Chin J Rehabil Theory Pract. (2018) 229:1485–8. doi: 10.3969/j.issn.1006-9771.2018.12.025

[31] Williams C Northstone K Harrad RA Sparrow JM Harvey I ALSPAC Study Team. Amblyopia treatment outcomes after screening before or at 3 years: follow up from randomised trial. BMJ. (2002) 324:1549. doi: 10.1136/bmj.324.7353.1549, PMID: 12089090 PMC116606

[32] Ding J Levi DM. Rebalancing binocular vision in amblyopia. Ophthalmic Physiol Opt. (2014) 34:199–213. doi: 10.1111/opo.12115, PMID: 24417338 PMC4072503

[33] Yuan Y Peng X Zhang M. Study on the frequency of ciliary muscle training for the prevention and control of myopia in physical education. Sport Sci Res. (2022) 43:19–23. doi: 10.12064/ssr.20220303

[34] Chu R. Key points for prevention and control of myopia in children. China Glass Sci Technol Mag. (2014) 50:6–8. doi: 10.3760/cma.j.issn.0412-4081.2014.01.003, PMID: 24709126

